# Supplementary figures and images for: The cancer angiogenesis co-culture assay: In vitro quantification of the angiogenic potential of tumoroids
Source: PLoS One. 2021 Jul 7;16(7):e0253258. doi: 10.1371/journal.pone.0253258 (PMC8263287; doi:10.1371/journal.pone.0253258)

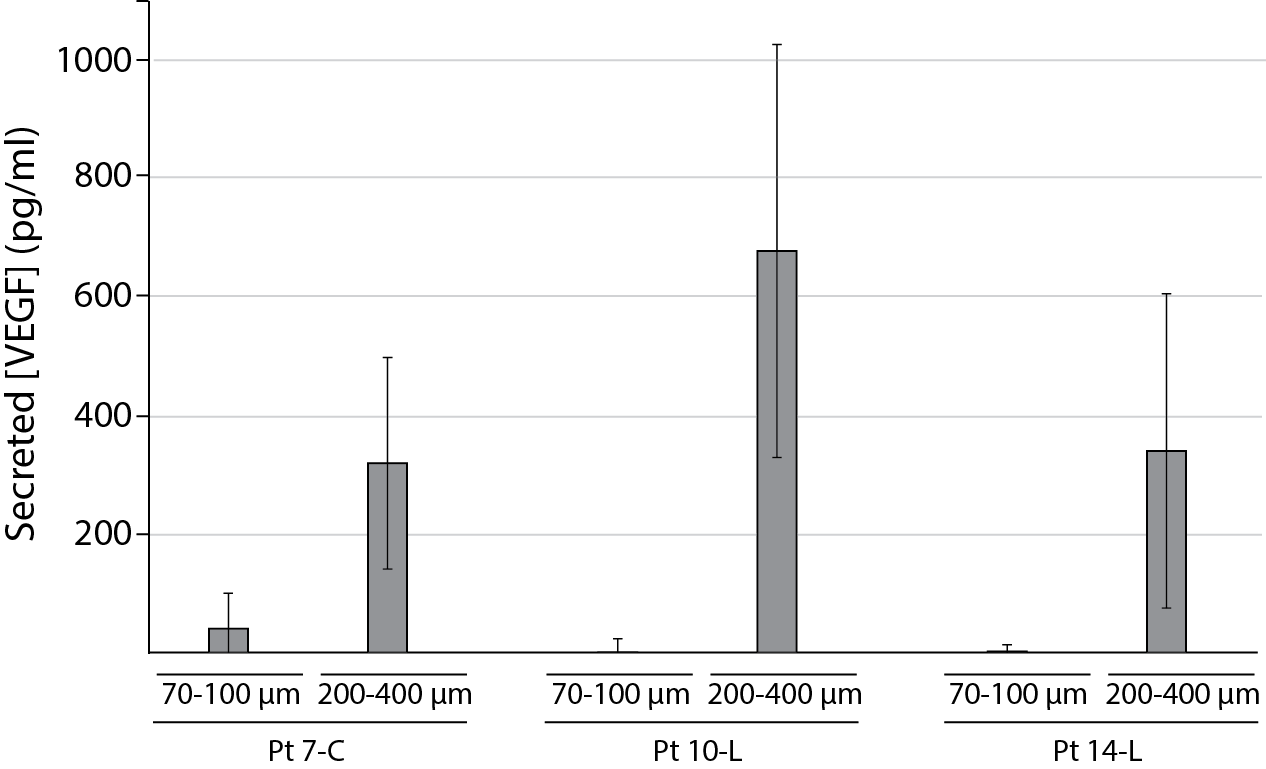

Supplement: S1 Fig — Tumoroids sized 70–100 μm (n = 40) and 200–400 μm (n = 10) were seeded in a 96-well plate and conditioned media were collected following 7 days of culture to measure the level of tumoroid-secreted VEGF by ELISA. Data are the average of triplicate samples ± SD. (TIF) [file pone.0253258.s001.tif]

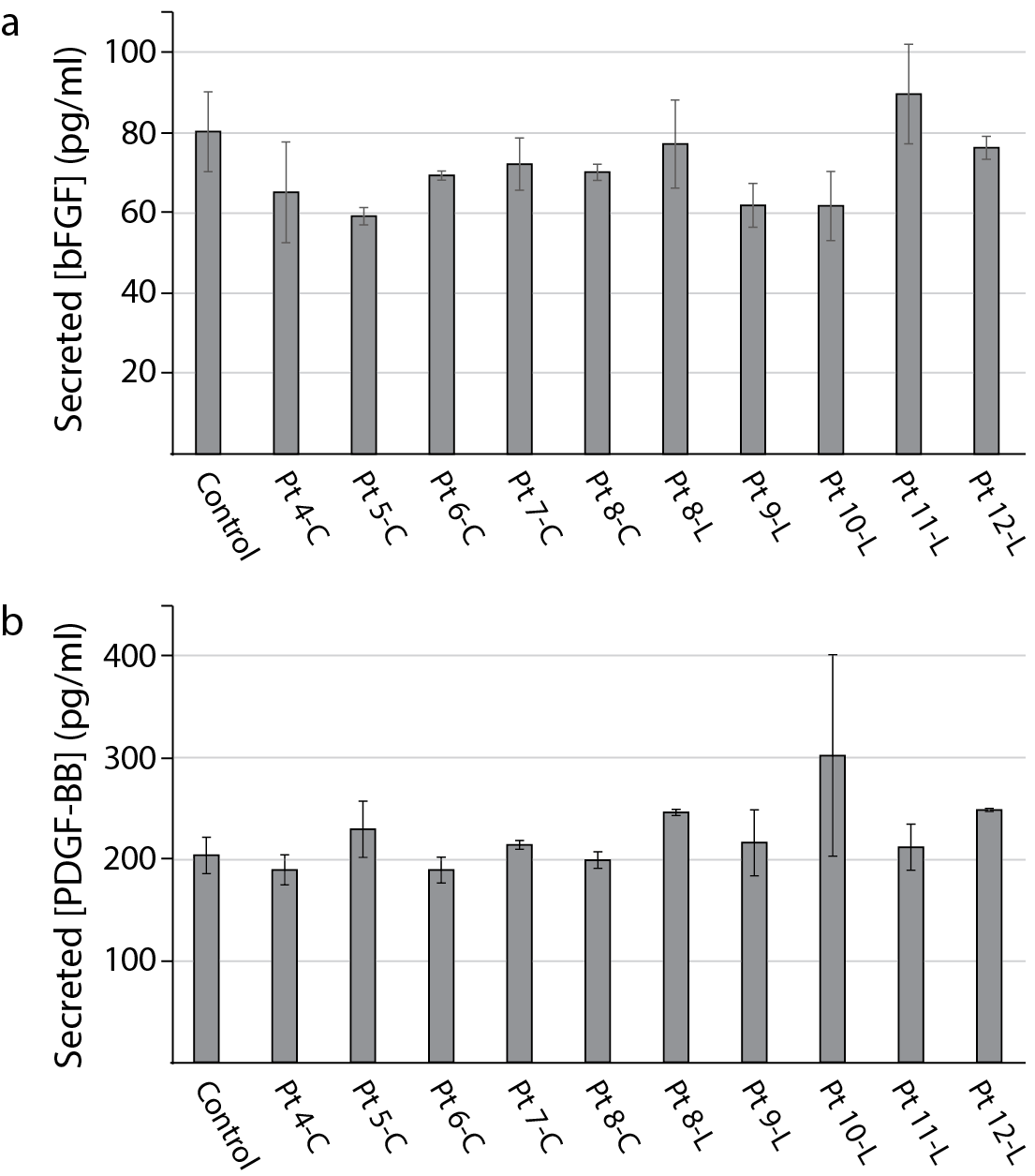

Supplement: S2 Fig — (a) Tumoroid-secreted bFGF measured in culture medium collected from the CACC assay and assayed for bFGF by ELISA. Data are the average of triplicate samples ± SD. (b) Tumoroid-secreted PDGF BB measured in culture medium collected from the CACC assay and assayed for PDGF BB by ELISA. Data are the average of duplicate samples ± SD. (TIF) [file pone.0253258.s002.tif]

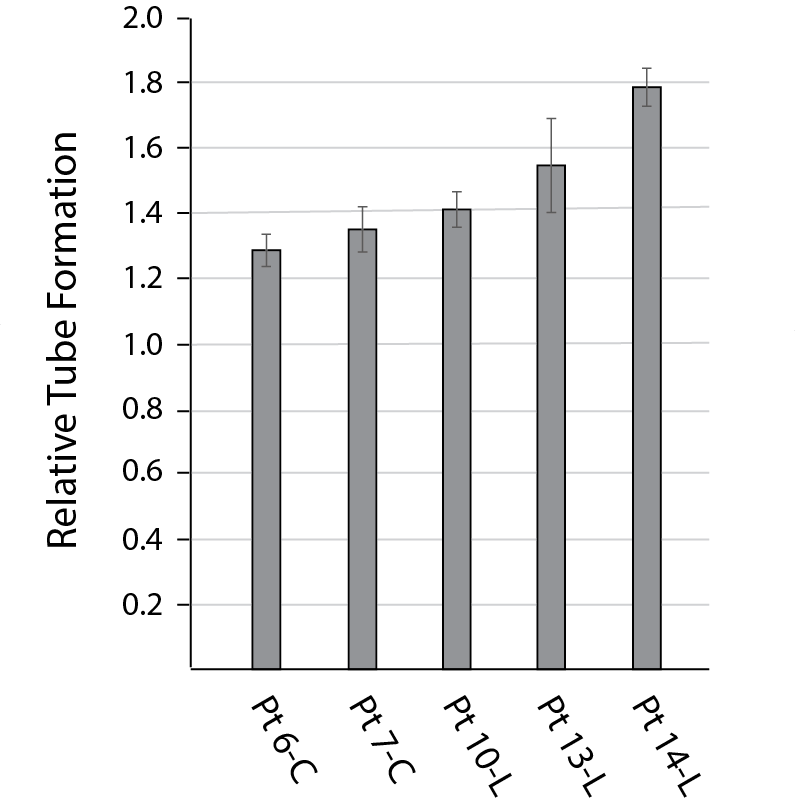

Supplement: S3 Fig — Tumoroids were introduced to the CACC assay on day 7. At the end of the experiment (day 11) the co-cultures were fixed and stained for the endothelial marker PECAM-1 to reveal the tubes. Tubes were quantified with the BioSense Solutions and 2cureX tube algorithm. Tube formation is expressed relative to control sample without tumoroids ± SD. Data are the average of 4 independent experiments for Pt 6-C, Pt 7-C and Pt 10-L, and 3 independent experiments for Pt 13-L and 14-L, with triplicate samples in each independent experiment. (TIF) [file pone.0253258.s003.tif]
