# Supplementary material for: The cancer angiogenesis co-culture assay: In vitro quantification of the angiogenic potential of tumoroids
Source: PLoS One. 2021 Jul 7;16(7):e0253258. doi: 10.1371/journal.pone.0253258 (PMC8263287; doi:10.1371/journal.pone.0253258)
Supplement: Attachment — Submitted filename: Response to reviewers.docx [file pone.0253258.s004.docx]

**Response to reviewers**

We thank for the thorough review of our manuscript.

*(all references to page and line numbers are from the revised manuscript with track changes)*

**Reviewer 1.**

*1) This assay does not measure sprouting angiogenesis as stated multiple times in the manuscript. This is a tubulogenesis assay. Sprouting angiogenesis is new blood vessel sprouting from pre-existing vessels. As stated in the discussion (page 19), Bevacizumab was designed to inhibit sprouting angiogenesis. The manuscript and its title should be modified to make this distinction clear. Furthermore, it is important to address the possibility that the lack of correlation with VEGF levels maybe because this is not an assay of VEGF-dependent sprouting angiogenesis.*

**We agree that the Matrigel chord assay is a tubulogenesis assay, where the cells coalesce in a very short amount of time. In the co-culture assay there is some coalescence early on, but then most of the assay is extension of sprouts and branching of existing structures formed early on. And each chord is capped by a tip cell that clearly leads migration and is morphological indistinguishable from the physiological equivalent. Several published studies have shown that the co-culture assay of angiogenesis recapitulates the key aspects of angiogenesis (see reference [36-38,40] in the manuscript). We have updated the introduction and discussion to reflect that this is not a *sprouting* angiogenesis assay. (p.5 l.93-94, p.20 l.451)**

*2) Importantly the nature of the assay (discussed in point 1 above) is relevant to the extensive discussion and clinical relevance of “HGP” on pages 19 – 20.*

**Thank you for pointing out that the HGP and MVD discussion was perhaps a bit to extensive. We have de-emphasised HGP (and removed MVD as it didn’t add much value) in the discussion and focused on how HGP could explain the non-angiogenic tumoroid cultures. (p.20-21 l.456-462)**

*3) HUVECs have a very high propensity to form tubes when plated as described. This may account for the small differences seen upon the addition of tumoroids, and perhaps the low sensitivity of this assay.*

**We have included a discussion of why HUVECs were chosen for our assay in the manuscript. (p19 l.425-431)**

*4) The data presented in most figures are the average of triplicate samples and not the average of multiple experiments. The validity of the results would be greatly enhanced if the experiments were performed more than once. This is particularly relevant since the magnitude of the effects are relatively small.*

**We have included supplementary figure 3 in which the Relative Tube Formation of the same five tumoroid cultures were tested independently 3-4 times. We believe these experiments demonstrate the reproducibility of the assay. (S3 Fig, p.19 l.430-431**

*5) The various patient-derived tumoroids appear to have a range of sensitivity to Bevacizumab in vitro. It would be very valuable to correlate these observations with actual patient-responsiveness information, if accessible.*

**We agree that correlating the CACC results with patient response would be extremely interesting. The patients included in this study were, however, curatively resected and therefore not offered anti-angiogenic treatment. We hope that such a prospective clinical trial can be performed in the near future. We have updated the discussion explaining why a correlation was not performed. (p.23 l.527-528)**

*6) An analysis (or at least a discussion) of the likely targets of the other TKIs tested should be provided.*

**We have included more information regarding the tested TKIs and their targets in both the introduction and discussion. Additionally, we have investigated the level of tumoroid secreted PDGF-BB. PDGF-BB is the highest-affinity ligand of PDGFR-β, which is a common target of the three investigated TKIs. (p4 l.74-76, p.15 l.334-338, p.22 l.493-501, p.22 l.503-511, p.23 l. 524-526, S2 Fig.)**

**Reviewer 2.**

*This study is attempting to model the tumor/vasculature microenvironment with tumoroids. The authors examine tube formation of HUVEC induced by patient tumoroids. Surprisingly, they observe no correlation between secreted VEGFA levels and tube formation but still a strong inhibitory effect of Avastin on tube formation across the majority of tumoroids. My main concern is the failure to fully optimize the tumoroid/endothelial assay prior to performing these anti-angiogenic drug screens. Other types of human endothelial cells should be tested before defaulting to umbilical veins cells. Ideally primary endothelial cells would be isolated from resected tumors, but at a minimum, HMEC and other human endothelial lines should be compared. This would greatly strengthen this reviewers confidence in the fidelity of the model to mimic the tumor micro-environment.*

**In the current CACC assay we decided to use an EC cell line to decrease the potential variance that could arise from using ECs extracted from tumour tissue. The aim of this study was to create an assay capable of robustly detecting differences between cancer cells. By using an EC cell line, we feel we have removed a source of variation. Furthermore, HUVECs are widely available and widely used in similar assays. With that being said we agree with the reviewer that testing both primary tumour associated ECs and other EC cell lines would be very interesting, but beyond the scope of this manuscript. We hope to perform a study where the same tumoroids are tested with different sources of ECs both primary and established cell lines and publish these results in a separate paper.**

**We have included a discussion of why HUVECs were chosen for our assay in the manuscript. (p19 l.425-431)**

**Reviewer 3.**

*1. While developing the co-culture assay, information about cell ratio (HUVECs: fibroblasts:tumoroids) missing. Also, what is the composition of the media used for co-culture assay?*

**We believe we have covered this in the material and methods section (Cancer angiogenesis co-culture assay) already. (p.9-10 l.195-215)**

*2. Did the necrosis core or hypoxia play any role in secretion of VEGF? Did the size of tumor spheroids play any role in tube formation?*

**An excellent question. We have not been able to perform the experiments needed to demonstrate hypoxia or apoptosis as a function of tumoroid size. We have included references to papers showing reduced oxygen in the center of larger spheres and included supplementary figure 1 evaluating the VEGF secretion from small 70-100 µum and large 200-400 µm tumoroids. The results show an average 35 fold increase in VEGF secretion between the small and large tumoroids. This to us indicates that the larger tumoroids are indeed experiencing hypoxia. (S1 Fig., p.14 l.295-298)**

*3. When developing CACC assay, were the size of tumor spheroids taken into consideration?*

**See question 2 above. (S1 Fig., p.14 l.295-298)**

*4. Fig 2a: Fluorescent images of all conditions need to be provided*

**Figure 2 has been updated to show representative images for each VEGF concentration.**

*5. For investigating the efficacy of angiogenesis inhibitors, why comparison was made with 3.75 ng/ml VEGF treated samples?*

**The VEGF concentration of 3.75 ng/ml was selected as this is approximately the highest concentration of VEGF we observed secreted by our tumoroids cultures. We have updated the Results section to explain this. (p.13 l.269-270)**

*6. Why only the concentrations of VEGF and bFGF in the conditioned media was tested? Specially, when no correlation between high VEGF concentration and tubule formation was observed in Fig 5. Concentrations of other pro-angiogenic molecules released by cancer cells need to be evaluated.*

**We have included a figure with the bFGF secretion results and added ELISA measurements of PDGF-BB levels from the tumoroid cultures (supplementary figure 2) as we believe these three (VEGF, bFGF and PDGF-BB) are key factors in angiogenesis and some of the most relevant ligands targeted by the anti-angiogenic compounds investigated in this manuscript. The list of potentially relevant factors is very long, and it is beyond the scope of this manuscript to investigate them all. (S2 Fig., p.15 l.334-338, p.22 l.493-500)**

*7. Most of the anti-angiogenic agents tested are multi-kinase inhibitors. The concentrations of the corresponding growth factors need to be evaluated.*

**See question 6 above. Furthermore, it would be very interesting to try to discern the involved signalling pathways using more specific kinase inhibitors, however, this is unfortunately beyond the scope of this manuscript. (S2 Fig., p.15 l.334-338, p.22 l.493-500)**

**Financial Disclosure**

SLBT and NM was supported by the Innovation Fond Denmark  (<http://innovationsfonden.dk/en>), grant no. 5184-00101B. SLBT received funding from Agnes & Poul Friis Fund (grant no. 81008-001), C.C. Klestrup & hustrus Henriette Klestrups Mindelegat (grant no. 0660-001), Carl & Ellen Hertz legat til dansk læge- og naturvidenskab (grant no. 7179-2), The Drost Fundation (grant no. 12120-1), and Familien Hede Nielsens Fund (grant no 2017-0423).

The funders provided support in the form of salaries for authors SLBT and NM but did not have any additional role in the study design, data collection and analysis, decision to publish, or preparation of the manuscript. The specific roles of these authors are articulated in the ‘author contributions’ section.

SLBT, GH, OT, RS and JT are fulltime employees at 2cureX, Symbion, Denmark (<https://www.2curex.com/>). 2cureX provided support in the form of salaries for authors GH, OT, RS and JT. ES is a fulltime employee at BioSense Solutions, Farum, Denmark (<https://biosensesolutions.dk/>). BioSense Solutions provided support in the form of salaries for ES.

HW, LH, KQ, HH and HM received no specific funding for this work.

**Competing Interests**

I have read the journal's policy and the authors of this manuscript have the following competing interests:

SLBT, GH, OT, RS and JT are fulltime employees at 2cureX, Birkerød, Denmark.

GH and OT are founders and stockholders for 2cureX.

ES is a fulltime employee at BioSense Solutions, Farum, Denmark.

ES is a founder and stockholders for BioSense Solutions.

This does not alter our adherence to PLOS ONE policies on sharing data and materials.

NM, HW, LH, KQ, HH and HM declare no competing interests.
