# Supplementary material for: The cancer angiogenesis co-culture assay: In vitro quantification of the angiogenic potential of tumoroids
Source: PLoS One. 2021 Jul 7;16(7):e0253258. doi: 10.1371/journal.pone.0253258 (PMC8263287; doi:10.1371/journal.pone.0253258)
Supplement: Attachment — Submitted filename: Letter to the editor round 2.docx [file pone.0253258.s005.docx]

Copenhagen, May 2021

Dear Christina,

Thank you for the feedback to our manuscript. We have thoroughly reviewed our manuscript following the comments from reviewer 2. In our opinion the most “problematic” conclusion was in the abstract and final part of the discussion (line 525). We agree that the last sentence in the original abstract was a bit optimistic. Consequently, we have revised the manuscript and emphasized that a clinical verification is needed to prove the predictive value of our assay. We also point to the last portion of the Discussion (lines 529-533) where we also write that validation of the assay will require a clinical trial.

The field of angiogenesis is currently challenged by the fact that no combination of angiogenic factors and/or pathological measurements is able to predict patient response to anti-angiogenic treatment such as Bevacizumab. Consequently, verifying the validity of our assay against known angiogenic factors could prove an impossible task. The observation that our results do not correlate with individual angiogenic factors (such as VEGF) (just like in the clinic) leads us to speculate that our assay could potentially recapture some of the biological complexity of tumour angiogenesis. As written above and in our manuscript (and now emphasised in the updated abstract) the only way to verify this correlation is by conducting a clinical trial which is beyond the scope of this manuscript.

We hope that the changes and the above explanation is sufficient to satisfy you and reviewer 2.

On behalf of the authors,

Best regards,

Jacob Thastrup
